# Early Light-Inducible Protein (ELIP) Can Enhance Resistance to Cold-Induced Photooxidative Stress in *Chlamydomonas reinhardtii*

**DOI:** 10.3389/fphys.2020.01083

**Published:** 2020-08-25

**Authors:** Ji Woong Lee, Seung Hi Lee, Jong Won Han, Gwang Hoon Kim

**Affiliations:** ^1^Department of Biological Sciences, Kongju National University, Kongju, South Korea; ^2^Department of Applied Bioresource Science, National Marine Biodiversity Institute of Korea, Seocheon, South Korea

**Keywords:** ELIP, mutation, cold stress, ROS, CO_2_

## Abstract

Cold weather is one of the biggest challenges in establishing a large-scale microalgae culture facility in temperate regions. In order to develop a strain that is resistant to low temperatures and still maintains high photosynthetic efficiency, transgenic studies have been conducted targeting many genes. Early light-inducible proteins (ELIPs) located in thylakoid membranes are known to protect photosynthetic machinery from various environmental stresses in higher plants. An *ELIP* homolog was identified from *Chlamydomonas reinhardtii* and named *ELIP3*. The role of the gene was analyzed in terms of photosynthetic CO_2_ assimilation under cold stress. Western blot results showed a significant accumulation of ELIP3 when the cells were exposed to cold stress (4°C). High light stress alone did not induce the accumulation of the protein. Enhanced expression of ELIP3 helped survival of the cell under photo-oxidative stress. The influx of CO_2_ to the photobioreactor induced strong accumulation of ELIP3, and enhanced survival of the cell under high light and cold stress. When the oxidative stress was reduced by adding a ROS quencher, TEMPOL, to the media the expression of ELIP3 was reduced. A knockdown mutant showed much lower photosynthetic efficiency than wild type in low temperature, and died rapidly when it was exposed to high light and cold stress. The overexpression mutant survived significantly longer in the same conditions. Interestingly, knockdown mutants showed negative phototaxis, while the overexpression mutant showed positive phototaxis. These results suggest that ELIP3 may be involved in the regulation of the redox state of the cell and takes important role in protecting the photosystem under photooxidative stress in low temperatures.

## Introduction

Exposure to high light and cold stress causes the accumulation of reactive oxygen species (ROS) in plant cells, and plants have evolved various ROS scavenging machineries (Caverzan et al., 2012; Choudhury et al., 2013). Some plants also actively produce ROS and use it as a cell signaling molecule for development or defense against pathogen attack (Im et al., 2019). Most ROS in plant cells are generated by electrons originating from photosystem I (PSI), which generates superoxide anion (O_2_^–^) from molecular oxygen followed by the increase of hydrogen peroxide (H_2_O_2_) by superoxide dismutase (SOD) activity (Apel and Hirt, 2004; Asada, 2006). In low temperature, the functioning of the Calvin cycle declines, in part, due to a decrease in the activity of the CO_2_ assimilation enzyme ribulose-1,5-bisphosphate carboxylase/oxygenase (Rubisco) (Allen and Ort, 2001; Hirotsu et al., 2004; Zhou et al., 2004; Jiang et al., 2013; Gan et al., 2019). The limitation of photosynthetic CO_2_ fixation causes over-reduction of the photosynthetic electron transport chain and promotes singlet oxygen (^1^O_2_) generation by excited triplet state of chlorophyll in the antenna, as well as the reaction center of photosystem II (PSII) (Krieger-Liszkay et al., 2008). ROS generated in chloroplasts is usually scavenged by the water-water cycle (Asada, 1999), but ROS overproduction due to a combination of high light and low temperature can inhibit the turnover of the D1 protein of the PSII reaction center and lead to photoinhibition (Powles, 1984; Allen and Ort, 2001; Choi et al., 2002; Takahashi and Murata, 2006, 2008). In addition, overproduction of ROS in low temperature not only leads to oxidative inactivation of Calvin cycle enzymes, but also irreversible oxidative damage to chloroplast, and consequently severely inhibits photosynthesis and growth of plants (Kaiser, 1976; Tanaka et al., 1982).

Microalgae are biological resources that can produce not only renewable fuel, but also various products such as feedstock, food, materials, and useful chemicals (Zhu, 2015; Ruiz et al., 2016). Microalgae are considered as an optimal biomass producer as they can consume less water, and arable land, than land crops, but large-scale microalgal production is mostly carried out in tropical or sub-tropical locations where strains are grown at water temperatures between 15–30°C as growth is inhibited at low temperatures (Spolaore et al., 2006; Chisti, 2007). For microalgal production in cold climates, photobioreactor housed within greenhouses with additional heating systems are necessary (Baliga and Powers, 2010; Laamanen et al., 2014). Development of genetically engineered strains with reduced energy consumption has been extensively exploited for the optimal year-round production of various microalgae (Rosenberg et al., 2008; Radakovits et al., 2010; Apel and Weuster-Botz, 2015; Kotchoni et al., 2016). However, little is known of the genes involved in low temperature tolerance in microalgae.

Early light-inducible proteins (ELIPs) belong to the light-harvesting complex (LHC)-like proteins with chlorophyll-binding motifs, which has been extensively studied in land plants as a target gene to enhance tolerance against high light stress (Grimm and Kloppstech, 1987; Adamska, 2001; Heddad and Adamska, 2002; Engelken et al., 2012). In land plants, ELIPs were induced by light stress (high light, blue light, UV-A), and in a light intensity-dependent manner, controlled by the blue/UV light photoreceptor cryptochrome1 (CRY1) (Adamska et al., 1992a, b; Kleine et al., 2007). ELIP1 and ELIP2 of *Arabidopsis thaliana* are associated with the monomeric and trimeric major LHCb antenna of PSII and are considered to have a protective function for chloroplast from photooxidative stress under high light (Hutin et al., 2003; Heddad et al., 2006). However, in a study using *elip1*/*elip2* mutants, the possibility that ELIPs modulate the xanthophyll cycle and have a photo-protective function only in severe stress conditions was proposed (Rossini et al., 2006). In the same vein, ELIPs were most highly expressed when photooxidative stress was aggravated due to combined stresses of high light and low temperature (Shimosaka et al., 1999; Fowler and Thomashow, 2002; Casazza et al., 2005; Han and Kim, 2013).

*Chlamydomonas reinhardtii* is one of the major target species for biomass production, but year-round production is limited in temperate climates as photosynthetic capacity decreases below 15°C and cell death occurs at 3°C (Hema et al., 2007; Kotchoni et al., 2016; Kwak et al., 2017). Among 10 *ELIP* homologs reported in *C. reinhardtii*, only *ELIP3* has the conserved LHC motifs on the transmembrane domains and shows light-induced expression (Elrad and Grossman, 2004; Teramoto et al., 2004; Zones et al., 2015; Rochaix and Bassi, 2019). In this study, we characterized the function of *ELIP3* in photooxidative stress conditions and evaluated the utility of the gene as a target for the development of low temperature-tolerant strains.

## Materials and Methods

### Algal Culture Conditions and Stress Treatment

*Chlamydomonas reinhardtii* CC-125 originating from the Chlamydomonas Resource Center^1^ was used as the wild type (WT). WT and transgenic strains were cultured in Tris–acetate-phosphate (TAP) medium and agar plates (Harris., 2009) at 23°C under 50 μmol photons m^–2^ s^–1^ (12 L: 12 D). White fluorescent lamps (Osram FL-60 SW, South Korea), high-light LED (CR-PAR30, CR-LED, China) and UV lamps (G15T8E; F15T8BL, Sankyo Denki, Japan) were used for light conditions. Culture chambers (Vision Biotech, South Korea) at each temperature (4, 10, 23°C) were used for temperature experiments. *C. reinhardtii* were exposed to various combinations of irradiance and temperature at a cell concentration of 5 × 10^6^ cells/ml. CO_2_ experiments were performed using a custom-made photobioreactor with a water-jacket (Pyrex 500 ml, Scilab, South Korea) for low temperature. For CO_2_ treatments, high quality 5% CO_2_ was obtained from Special Gas Company (Daejeon, South Korea). CO_2_ experiments were performed following a previously described method (Han and Kim, 2013).

### Western Blot Analysis

Western blotting was performed to analyze the accumulation of ELIP3 under stress conditions. *C. reinhardtii* (10^7^ cells) was collected using centrifugation at 11,000 *g* for 10 min. The pellet was resuspended with 1× Laemmli buffer (S3401, Sigma-Aldrich, United States) and incubated at 100°C for 5 min, then centrifuged at 11,000 *g* for 10 min to obtain the supernatant. The supernatant was loaded onto 12% (w/v) SDS-polyacrylamide gels and transferred to polyvinylidene fluoride (PVDF) membranes (Bio-Rad, United States) using a Mini Trans–Blot® Cell (Bio-Rad, United States). The membrane was blocked with TBS buffer (Tris–buffered saline) with 5% skim milk and 0.1% Tween 20 with agitation. For the detection of ELIP3 protein, we developed rabbit polyclonal antibodies raised against the antigenic sequence, FGKSYTPEEWEKEVASGAF derived from the ELIP3 (XM_001694629) sequence (Ab Frontier, South Korea). The blots with expected protein size were incubated in primary Anti-ATPβ (loading control, 1:3000, Agrisera, Sweden), Anti-psbA (D1 protein) (1:5000, Agrisera, Sweden), or Anti-ELIP3 (1:2000) at 4°C for 12 h. We used less diluted Anti-ELIP3 (1:1500) when we perform western blotting at 10 and 23°C to enhance sensitivity. Then, the blots were incubated with horseradish peroxidase (HRP)-conjugated goat anti-rabbit secondary antibodies (Ab Frontier, South Korea) for 1 h. Protein levels were detected using a chemiluminescence kit (Amersham, United Kingdom). The relative protein levels were quantified using Quantity One software (Bio-Rad, United States) and normalized by ATPβ levels.

### Generation of Transgenic Strains

For the overexpression strain of *ELIP3*, a cloned coding region of *ELIP3* was inserted into the pChlamy_3/D-TOPO vector (Invitrogen, United States) (Supplementary Figure S1). The linearized plasmid, using the restriction enzyme *Sca*I, was transformed into the CC-125 strain by GenePulser Xcell electroporation system (Bio-Rad, United States). For the RNAi expression vector, the partial coding region (314 bp) of ELIP3 was amplified from cDNA using the specific primers ELIP3_RNAi-F and ELIP3_RNAi-R (Supplementary Table 1). The fragment of ELIP3 was added *Pst*I and *Xba*I restriction enzyme sites in the 5′ end and *Bam*HI and *Hin*dIII restriction enzyme sites in the 3′ end by the extra base pairs on the 5′ end of the primers. Using these enzyme restriction sites, fragment was ligated sequentially to Spacer (Rohr et al., 2004) and pCr102 vector (Kim et al., 2011), which generated a hairpin RNA construct from the 3′ UTR of *aph7”* (Supplementary Figure S1). The constructed vector was then transformed into CC-125 by electroporation after linearization using *Kpn*I. Electroporated cells were recovered overnight in TAP medium containing 40 mM of sucrose, with agitation (120 rpm) under dim light (Shin et al., 2016). The transgenic strains were selected from TAP agar plates containing hygromycin B (15 mg/l).

### Treatment of Redox Reagents

50 ml (5 × 10^6^ cells/ml) of *C. reinhardtii* cells were centrifuged, and the pellets were resuspended in each reagent containing TAP media. Hydrogen peroxide (H_2_O_2_, Sigma-Aldrich, United States), norflurazon (NF, Sigma-Aldrich, United States) and 4-hydroxy-2, 2, 6, 6-tetramethylpiperidine 1-oxyl (TEMPOL, Sigma-Aldrich, United States) were used as the redox reagents.

### Measurement of Chlorophyll Fluorescence Parameters

To measure the photosynthetic efficiency of WT and transgenic strains at different temperatures a previously described method was used (Han and Kim, 2013). Chlorophyll fluorescence measurements were made using a PHYTO-PAM (Walz, Germany). The *C. reinhardtii* cells (5 × 10^6^ cells/ml) of each strain were incubated at 23 or 4°C in the light intensity of 50 μmol photons m^–2^ s^–1^ and 1 ml was harvested for calculation of fluorescence parameters. The effective and maximum quantum yields, and rapid light curves (RLC) were measured at designated times after exposure (Ralph and Gademann, 2005). RLCs were constructed based on nine actinic increasing light levels (0, 16, 64, 128, 192, 320, 512, 832, 1088, 1344 μmol photons m^–2^ s^–1^). The effective and maximum quantum yield of PSII were calculated as ΦPSII = Δ*F*/*F*_m_′ = (*F*_m_′-*F*)/*F*_m_′ and *F*_v_/*F*_m_ = (*F*_m_-*F*_0_)/*F*_m_, respectively. *F*_m_′ and *F* values were measured in light incubated strains, and *F*_m_ and *F* values were measured by dark-adaptation for 10 min (Ralph and Gademann, 2005). The relative electron transport rate (rETR) was calculated as ETR = 0.84 × 0.5 × ΦPSII × light intensity for each light level. Due to the occurrence of *F*_m_′ values higher than the *F*_m_ value measured after dark-adaptation, non-photochemical quenching (NPQ) values were calculated as NPQ = (*F*_m_′_max_-*F*_m_′)/*F*_m_′ (Serôdio et al., 2007).

### Quantitative PCR and Northern Blot Analysis of *ELIP3*

Total RNA was isolated using the RNeasy Plant Mini Kit (Qiagen, Germany). The RNA concentration was determined using a spectrophotometer (MaestroNano, MaestroGen, Taiwan) and its integrity was assessed by 1.2% agarose-formaldehyde gel electrophoresis. First strand cDNAs were synthesized using Accuscript High Fidelity 1st strand cDNA kit (Agilent Technologies, United States). Real-time quantitative PCR was performed using QuantiSpeed SYBR Hi-Rox Kit (PhileKorea, South Korea) in a StepOnePlus Real-Time PCR System (Applied Biosystems, United States) as described previously (Lee and Kim, 2019). The primers used for qPCR are shown in Supplementary Table S1. The housekeeping gene 18S rRNA was used as an endogenous internal control to normalize gene expression. For northern blot analysis of *ELIP3*, the DNA probe was directly amplified and labeled with DIG-dUTP by PCR of the *ELIP3* genes from cDNA using the DIG probe Synthesis Kit (Roche, Germany). The primers used for probe synthesis are shown in Supplementary Table S1. Northern blotting was performed as described previously (Han and Kim, 2013). The relative mRNA levels were quantified using Quantity One software (Bio-Rad, United States) and normalized by band intensities of rRNA.

### Statistical Analysis

Statistical analysis was performed with GraphPad Prism 8 software (GraphPad Software, United States). The Shapiro-Wilk test was used to determine the distribution of the data. Statistical significance was determined by one-way Welch’s ANOVA with Dunnett’s T3 *post hoc* test or two-way ANOVA with Bonferroni’s *post hoc* test for multiple comparisons. In some cases, the two-tailed unpaired Student’s *t*-test (with Welch’s correction) was used for comparing two groups. Statistical significance was accepted when *P* < 0.05.

## Results

### Accumulation of ELIP3 in Response to Light and Low Temperature

**FIGURE 1:**
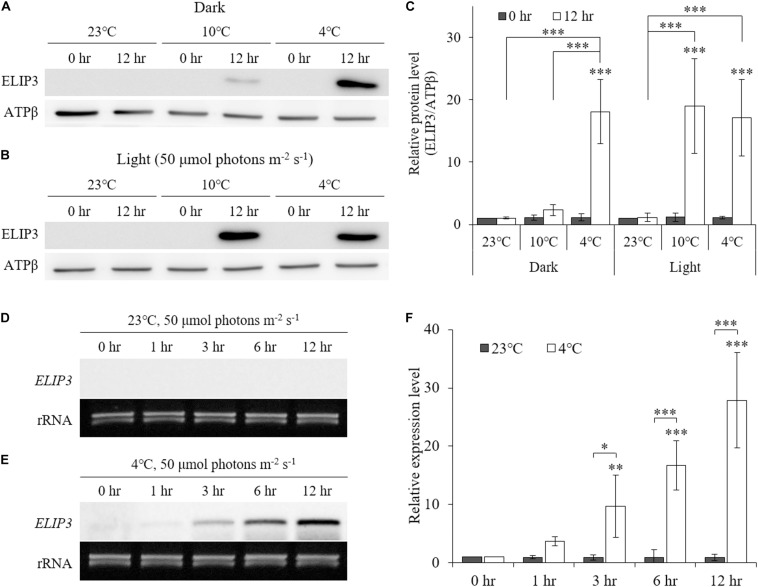
Western blot **(A, B)** and Northern blot **(D, E)** analysis showing accumulation of ELIP3 in different temperature regimes (23, 10, and 4°C). **(A)** Accumulation in the dark. **(B)** Enhanced accumulation in light (50 μmol photons m^–2^ s^–1^). **(C)** Densitometry of western blot results. Data are expressed as mean ± SD (two-way ANOVA with Bonferroni’s multiple comparisons test, *n* = 4). **(D)** Expression of *ELIP3* at 23°C. **(E)** Expression of *ELIP3* at 4°C. **(F)** Densitometry of northern blot results. Data are expressed as mean ± SD (two-way ANOVA with Bonferroni’s multiple comparisons test, *n* = 4). ATPβ was used as a loading control for western blotting, and rRNA for northern blotting. ^∗^*P* < 0.05, ^∗∗^*P* < 0.01, ^∗∗∗^*P* < 0.001.

**FIGURE 2:**
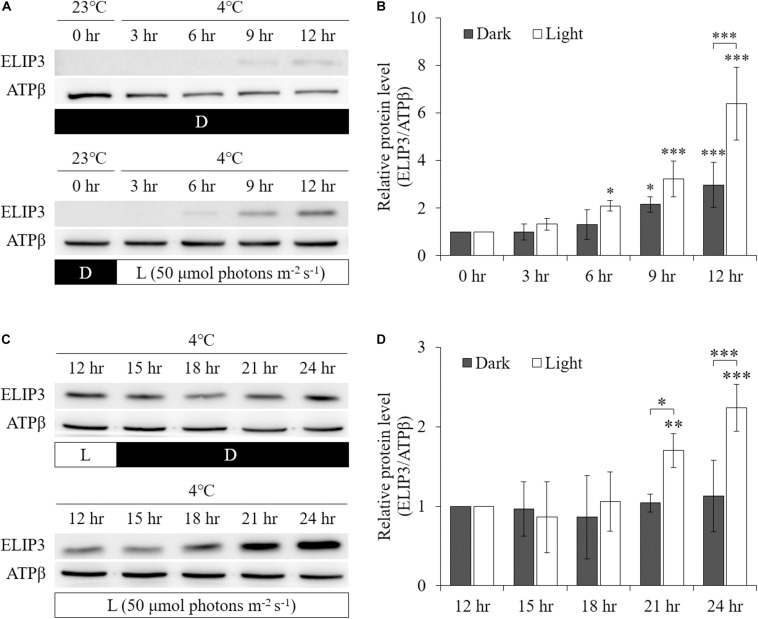
Western blot analysis showing accumulation of ELIP3 in response to low temperature (4°C) in dark and light (50 μmol photons m^–2^ s^–1^) conditions. **(A)** In dark, the accumulation started at 9 h. In light condition, the accumulation was stronger and started earlier at 6 h. **(B)** Densitometry of western blots in **(A)**. Data are expressed as mean ± SD (two-way ANOVA with Bonferroni’s multiple comparisons test, *n* = 3). **(C)** Transfer from light to dark or continuous light after 12 h of preincubation in light. Enhanced accumulation of ELIP3 in continuous exposure to light. **(D)** Densitometry of western blots in **(C)**. Data are expressed as mean ± SD (two-way ANOVA with Bonferroni’s multiple comparisons test, *n* = 3). ATPβ was used as a loading control. ^∗^*P* < 0.05, ^∗∗^*P* < 0.01, ^∗∗∗^*P* < 0.001.

Low temperature was found to enhance ELIP3 protein accumulation. For cells maintained in the dark, ELIP3 protein abundance was elevated after 12 h exposure to 4°C (Figures 1A,C). Further, ELIP3 protein abundance was elevated after 12 h exposure to light when the cells were incubated at both 10 and 4°C (Figures 1B,C). Northern blot analysis also showed a low temperature-dependent expression of *ELIP3* (Figures 1D,E). There was a time delay between the expression of *ELIP3* mRNA and accumulation of the protein. The expression of *ELIP3* was detected at 3 h after incubation at 4°C and gradually increased over time (Figures 1E,F), while the accumulation of the protein was observed from 6 h in light and from 9 h in the dark (Figures 2A,B). Once ELIP3 accumulated in light for 12 h, the amount of protein did not change even after the cells were transferred to dark for 12 h (Figure 2C). The accumulation of ELIP3 significantly increased from 21 h after exposure to light (Figures 2C,D).

**FIGURE 3:**
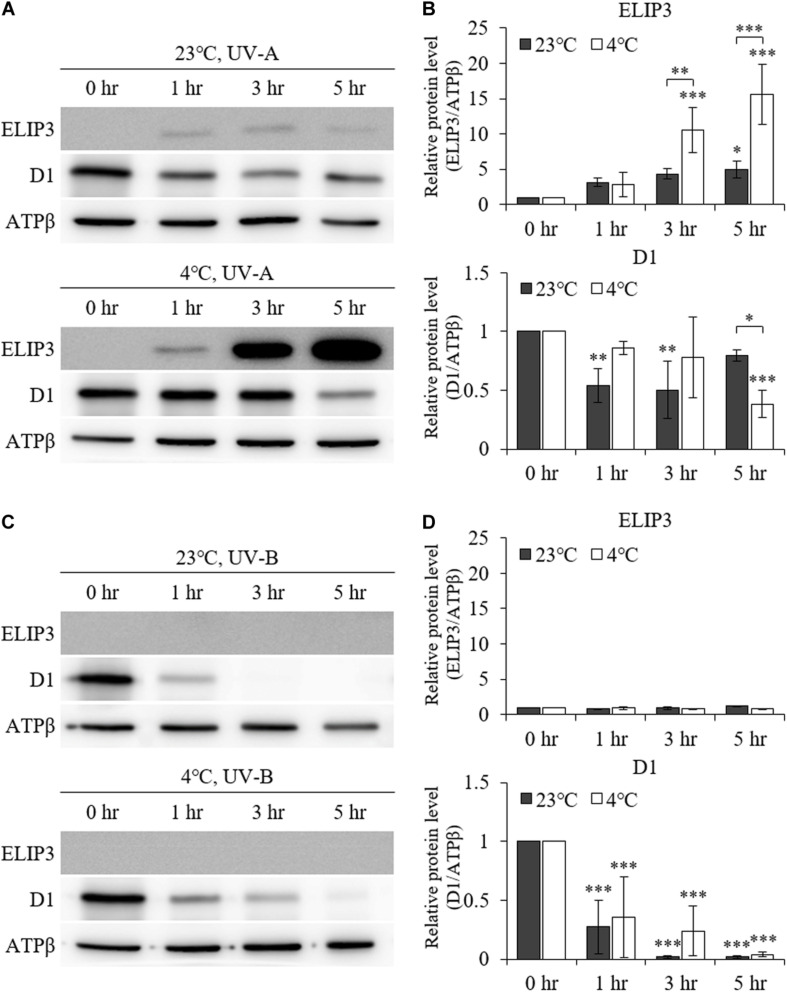
Western blot analysis under UV irradiation. **(A)** Accumulation of ELIP3 and D1 in UV–A irradiation. **(B)** Densitometry of western blots in **(A)**. Data are expressed as mean ± SD (two-way ANOVA with Bonferroni’s multiple comparisons test, *n* = 3). **(C)** Accumulation of ELIP3 and D1 in UV-B irradiation. **(D)** Densitometry of western blots in **(C)**. Data are expressed as mean ± SD (two-way ANOVA with Bonferroni’s multiple comparisons test, *n* = 3). ATPβ was used as a loading control. ^∗^*P* < 0.05, ^∗∗^*P* < 0.01, ^∗∗∗^*P* < 0.001.

UV–A irradiation increased ELIP3 protein abundance after 1 h at 23°C. Further, at 4°C, 3 h of UV–A irradiation markedly elevated ELIP3 protein abundance (Figures 3A,B). At warm temperature, the expression of D1, a reaction core protein of PSII, was decreased after 1 h of UV-A irradiation, while at 4°C, the expression of D1 was decreased after 5 h of UV-A irradiation (Figures 3A,B). UV-B irradiation did not induced accumulation of ELIP3 because of rapid cell death in this condition (Figures 3C,D).

**FIGURE 4:**
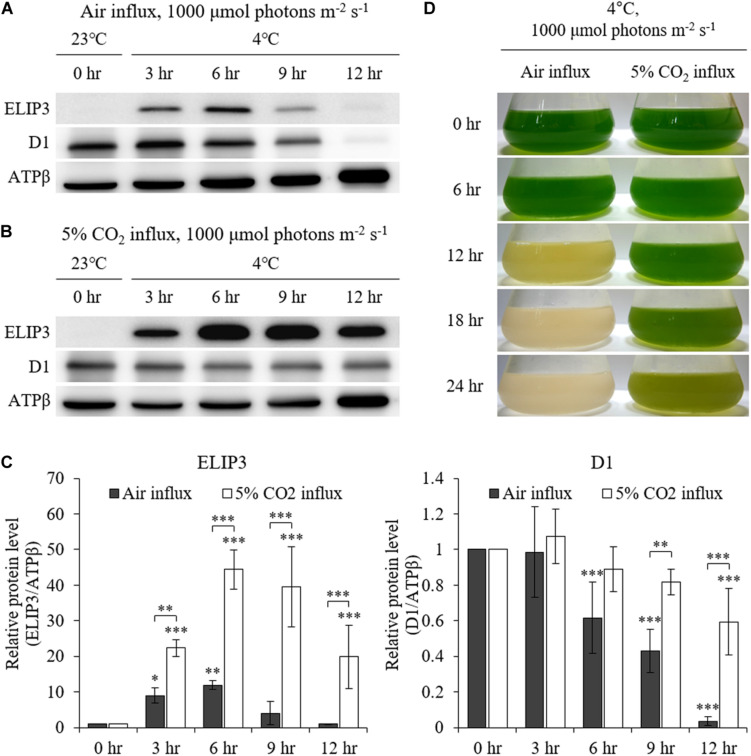
Western blot analysis of *C. reinhardtii* with 5% CO_2_ supply at low temperature (4°C) and high light (1,000 μmol photons m^–2^ s^–1^) using a photobioreactor. **(A)** Accumulation of ELIP3 and D1 in air flux condition. **(B)** Accumulation of ELIP3 and D1 with 5% CO_2_ supply. ATPβ was used as a loading control. **(C)** Densitometry of western blots in **(A)** and **(B)**. Data are expressed as mean ± SD (two-way ANOVA with Bonferroni’s multiple comparisons test, *n* = 3). **(D)** The response of *C. reinhardtii* cells to low temperature and high light with or without added CO_2_ supply. ^∗^*P* < 0.05, ^∗∗^*P* < 0.01, ^∗∗∗^*P* < 0.001.

The accumulation of ELIP3 was not induced in warm temperature even in very high light, 1000 μmol photons m^–2^ s^–1^, while accumulation of D1 was observed at the same condition (Figures 4A–C). When the cells were exposed to extreme high light (1,000 μmol photons m^–2^ s^–1^) at 4°C the cells lost color and began to die in 12 h (Figure 4D, left lane). ELIP3 and D1 protein disappeared at this time while the control protein ATPβ was still present (Figures 4A,C). When 5% CO_2_ gas was influxed to photobioreactor at the same condition cells survived much longer and chloroplasts managed to keep their color for 24 h (Figure 4D, right lane). Interestingly, the accumulation of ELIP3 was significantly enhanced with CO_2_ influx while the accumulation of D1 protein did not change much (Figures 4B,C). The effect of CO_2_ influx was not observed when the cells were exposed to moderate light, 50 μmol photons m^–2^ s^–1^, at 4°C (Supplementary Figure S2).

**FIGURE 5:**
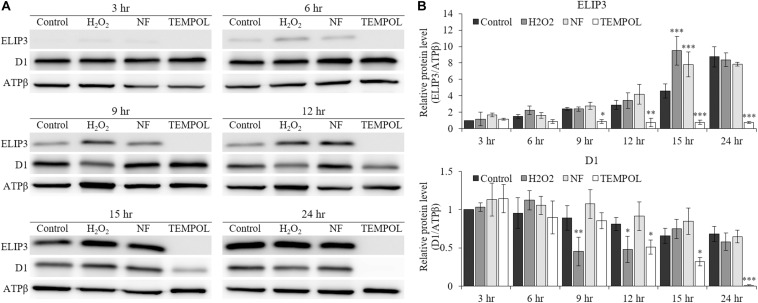
Western blot analysis showing effect of redox reagents on ELIP3 and D1 accumulation over time. **(A)** The cells were treated with H_2_O_2_ (50 μM), norflurazon (NF, 0.5 μM) and ROS quencher TEMPOL (20 mM) under low temperature (4°C, 50 μmol photons m^–2^ s^–1^). **(B)** Densitometry of western blots. Data are expressed as mean ± SD (two-way ANOVA vs control with Bonferroni’s multiple comparisons test, *n* = 3). ATPβ was used as a loading control. ^∗^*P* < 0.05, ^∗∗^*P* < 0.01, ^∗∗∗^*P* < 0.001.

The accumulation of ELIP3 and D1 protein in low temperature was affected by the treatment of redox reagents. When oxidative stress was enforced by H_2_O_2_ and carotenoid biosynthesis inhibitor, norflurazon (NF) treatment the accumulation of ELIP3 was enhanced in low temperature at 15 h after the treatments, but treatment with the ROS quencher, TEMPOL, completely blocked accumulation of ELIP3 for 24 h (Figures 5A,B). The accumulation of D1 protein was not affected by H_2_O_2_ treatment, instead it reduced gradually after TEMPOL treatment (Figure 5B).

### Characterization of Transgenic Mutants

**FIGURE 6:**
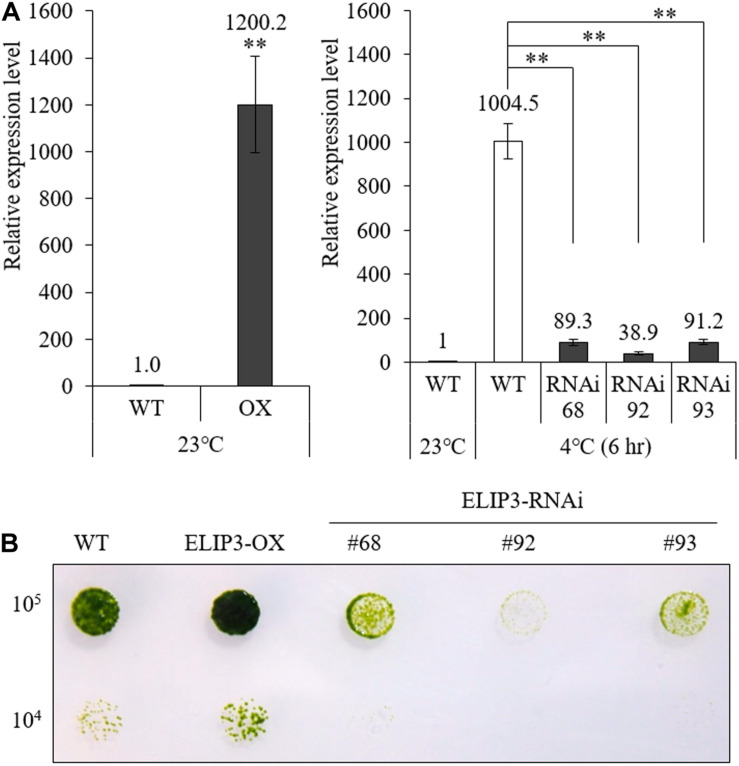
Expression of *ELIP3* in transgenic strains. **(A)** Transcription levels in *ELIP3* overexpression strain (OX) and knockdown strains (RNAi). Data are expressed as mean ± SD. (*t*-test and one-way ANOVA, ^∗∗^*P* < 0.01, *n* = 3). **(B)** Viability analysis using spot assay with TAP agar plate under low temperature. Cells of each strain (10^5^ cells/ml, 10^4^ cells/ml) were dispensed in the TAP agar plate and incubated at 10°C for 30 days.

We produced overexpression (OX) and knockdown (RNAi) mutants of *ELIP3*. Real-time PCR results showed that the expression of *ELIP3* was significantly higher in the OX mutant, in warm temperature, and much lower in RNAi strains than WT at 4°C (Figure 6A). Cell-blot assay of the mutants showed a significantly enhanced growth and viability in the OX strain compared to those in WT at low temperature (10°C), while the RNAi strains grew much slower and died earlier than WT in the same condition (Figure 6B).

**FIGURE 7:**
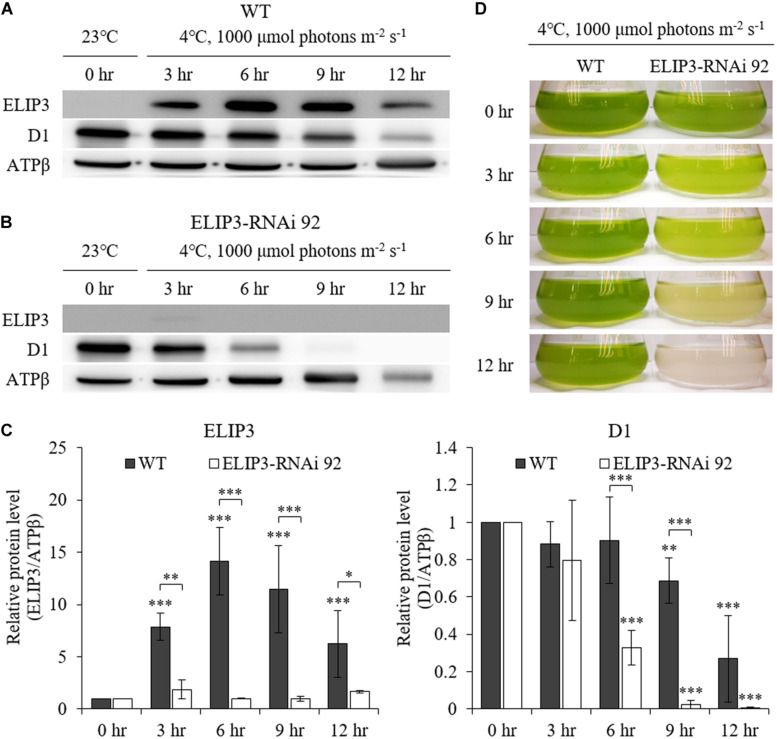
Characterization of ELIP3 knockdown strain RNAi 92 at low temperature (4°C) and high light (1,000 μmol photons m^–2^ s^–1^) conditions. **(A)** Western blot analysis of ELIP3 and D1 in WT and **(B)** RNAi 92. ATPβ was used as a loading control. **(C)** Densitometry of western blots in **(A,B)**. Data are expressed as mean ± SD (two-way ANOVA with Bonferroni’s multiple comparisons test, *n* = 3). **(D)** The response of WT and RNAi 92 strain to low temperature and high light in static culture condition. ^∗^*P* < 0.05, ^∗∗^*P* < 0.01, ^∗∗∗^*P* < 0.001.

Western blot analysis showed almost no accumulation of ELIP3 in the knockdown mutant, RNAi 92, at 4°C, 1000 μmol photons m^–2^ s^–1^ (Figures 7A,B). The quantitative densitometry showed a decrease of D1 levels in RNAi 92 compared to WT (Figure 7C). In static culture, the knockdown mutant began to lose pigment in less than 3 h at this condition and began to die in 9 h, while WT cultures were still green (Figure 7D).

**FIGURE 8:**
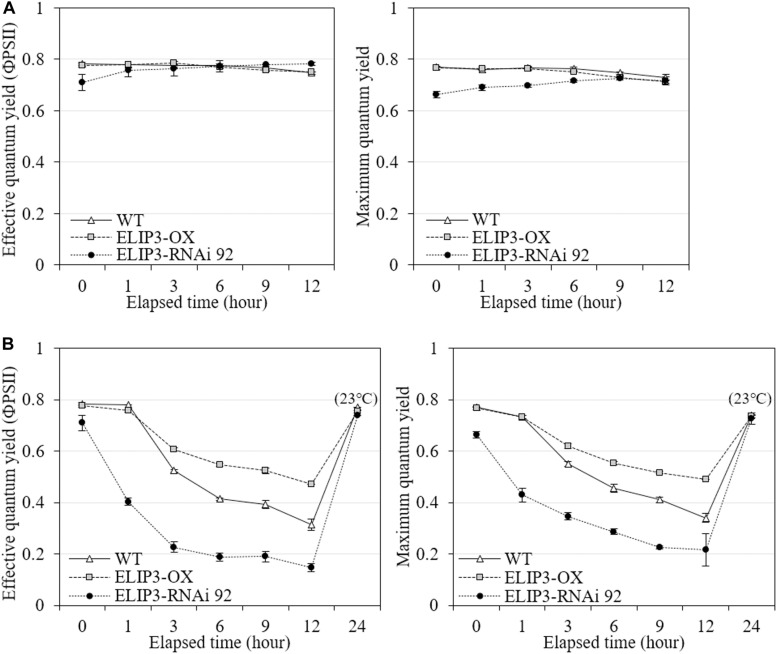
Effective quantum yield and maximum quantum yield measurements. Comparison of effective and maximum quantum yields from fluorescence measurement between WT, overexpression strain (OX) and knockdown strain (RNAi 92) at **(A)** 23°C and **(B)** 4°C. The strains measured at 4°C were incubated at 23°C under dark condition for 12 h, then recovery measured. Values represent the mean ± SD (*n* = 3).

**FIGURE 9:**
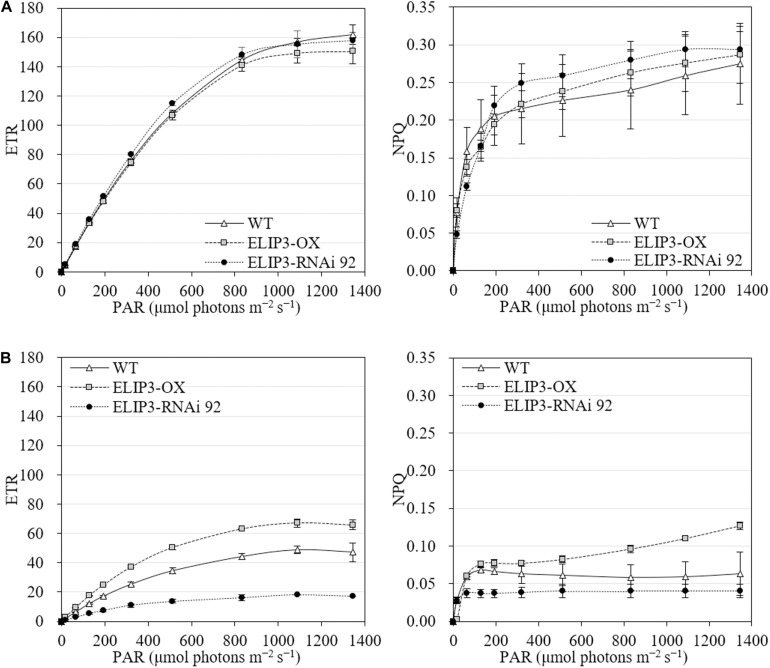
Electron transport rate (ETR) and non-photochemical quenching (NPQ) at different light levels for WT, overexpression strain (OX) and knockdown strain (RNAi 92). **(A)** 23°C. **(B)** 4°C. The values were measured after 12 h in each condition. Values represent the mean ± SD (*n* = 3).

The knockdown mutant, RNAi 92, showed lower values of effective quantum yield and maximum quantum yield compared to WT and OX strains initially in warm temperature, but the difference was recovered over time (Figure 8A), and the difference became significantly larger at 4°C (Figure 8B). The OX mutant showed the highest values at 4°C followed by WT. The effective and maximum quantum yield of knockdown mutant decreased more rapidly compared to WT and OX only for first 3 h (Figure 8B). When the samples were transferred back to 23°C, after 12 h, both quantum yield recovered in all strains (Figure 8). ETR and NPQ in photosynthetically active radiation (PAR) showed similar increasing curves in all strains at 23°C (Figure 9A). ETR saturation was reached much faster at 4°C, especially in the RNAi 92 strain (Figure 9B).

The transgenic mutants of ELIP3 showed a different phototaxis pattern. When knockdown mutant, RNAi 92, were exposed to unidirectional light, 50 μmol photons m^–2^ s^–1^ at 23°C, the cells showed negative phototaxis (Supplementary Video S1), while OX cells showed positive phototaxis like WT cells (Supplementary Video S2).

## Discussion

**FIGURE 10:**
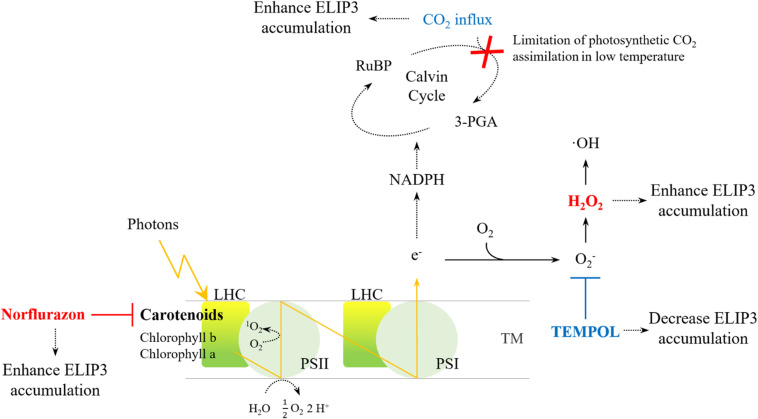
An overview of ROS generation in PSI and PSII and the effect of redox agents. The activity of the CO_2_ fixation enzyme, in the Cavin cycle, is limited under low temperatures, thereby leading to ROS production due to over-reduction of the photosystems. Increased CO_2_ may dissipate excess energy in the photosystems. The redox reagents were used to increase ROS (red) or to scavenge ROS (blue) in each photosystem. LHC, Light-harvesting complex; PS, photosystem; RuBP, Ribulose 1,5-bisphosphate; TM, thylakoid membrane; 3-PGA, 3-phosphoglyceric acid.

For optimal year-round production of microalgae in cold climates, development of a cold-tolerant strain is essential. The goal is to develop strains that grow well rather than simply survive in cold climates. In order to secure the growth rate of the cold-resistant strain, the genetic engineering of the strains has been extensively studied and some genes have been exploited as targets to maintain photosynthetic efficiency at low temperatures (e.g., Kotchoni et al., 2016). Our results suggest that *ELIP3* in *C. reinhardtii* may be a useful target gene to achieve these goals. The results from transgenic mutants showed that the overexpression of *ELIP3* not only enhances survival of cells, but also protects cells, to maintain photosynthetic efficiency in low temperature. Our results using redox reagents and CO_2_ influx suggested that cellular ROS generated in photooxidative stress may trigger accumulation of ELIP3. The expression of ELIP3 was significantly reduced when the oxidative stress was relieved with the treatment of antioxidant, TEMPOL, while the treatment with norflurazon or H_2_O_2_, the accumulation of ELIP3 was partially enhanced. CO_2_ influx to the media enhanced ELIP3 accumulation and relieved the limitation of CO_2_ assimilation in low temperature (Figure 10). Western blot analysis using antibody for the D1 protein suggested that ELIP3 is involved in mitigation of photooxidative stress in PSII also. These results suggest that ELIP3 of *C. reinhardtii* is involved in the mitigation of photooxidative stress at low temperature.

Algae appear to have higher thresholds for light stress than land plants. Microalgae grow optimally below 400 μmol photons m^–2^ s^–1^, and photoinhibition mostly occurs above 1,000 μmol photons m^–2^ s^–1^ (Singh and Singh, 2015). *C. reinhardtii* is usually cultured below 100 μmol photons m^–2^ s^–1^, but its best growth has been reported in much higher light intensity (Virtanen et al., 2019). Most land plants suffer from photo-oxidation around 400 μmol photons m^–2^ s^–1^ and the expression of ELIPs has been reported at even lower light intensity (Shimosaka et al., 1999). Most ELIPs reported in land plants accumulate in a high-light-dependent manner, and are not induced by low temperature alone, except for some *ELIP* homologs which can be induced also by other abiotic stresses (Adamska and Kloppstech, 1994; Ouvrard et al., 1996; Adamska, 2001). An *ELIP* homolog was reported in a multicellular green alga *Spirogyra varians*, which was also not induced by light but was induced by low temperature even in the dark (Han and Kim, 2013). It is not surprising that the accumulation of ELIP3 was induced by cold stress and not by light stress alone. Although the light intensity of 1,000 μmol photons m^–2^ s^–1^ was the highest level we can generate without raising the temperature in our apparatus, it might not be strong enough to generate photooxidative stress in *C. reinhardtii.* Cold stress especially when combined with high light may induce much stronger photooxidative stress to microalgae because cells are directly in contact with surrounding water body. ELIP3 in *C. reinhardtii* might have evolved to cope with the photooxidative stress in low temperature.

What is the signal that triggers accumulation of ELIP3 in *C. reinhardtii*? Our results from the experiments using redox agents showed that the accumulation of ELIP3 does not occur especially when cellular ROS level was scavenged with TEMPOL treatment. Cellular ROS are mostly generated in the photosystem. Under environmental stress, the increase of ROS by the photosystems acts as a major factor in photoinhibition (Murata et al., 2007; Lima-Melo et al., 2019). O_2_^–^, and subsequently H_2_O_2_, are generated by the Mehler reaction and water-water cycle from PSI, this reaction is an alternative electron sink that dissipated some excess excitation energy when CO_2_ availability is limited (Mehler, 1951; Asada, 1999; Takahashi and Murata, 2008). CO_2_-limitation promotes the production of singlet oxygen (^1^O_2_) in PSII, leading to the degradation of D1 and inhibition of the repair cycle in the reaction center along with H_2_O_2_, which is a major cause of photoinhibition (Nishiyama et al., 2001; Krieger-Liszkay, 2005; Takahashi and Murata, 2008). Although ROS production in environmental stress is genetically programmed for cellular signaling, high accumulation of ROS eventually leads to cell death (Shao et al., 2008; Choudhury et al., 2013; Mittler, 2017). It is well known that elevated CO_2_ supply can mitigate photooxidative stress in higher plants (Pérez-López et al., 2009; AbdElgawad et al., 2015; Xu et al., 2015). Previous studies in a multicellular green alga, *Spirogyra varians*, showed that CO_2_ influx may relieve photooxidative stress and reduce the expression of an *ELIP* homolog (Han and Kim, 2013). However, our results showed that CO_2_ influx in photooxidative conditions enhanced survival of the cell and induced greater accumulation of ELIP3.

Why does CO_2_ influx under photooxidative stress induce enhanced accumulation of ELIP3 in *C. reinhardtii*? It would have been more logical if the accumulation of ELIP3 is reduced as photooxidative stress was mitigated and cell survival was enhanced by CO_2_ influx, but the result was the opposite. It may be because we used mixotrophic TAP medium for the culture. It is known that these photomixotrophic conditions using TAP medium alleviate qE and photoinhibitory quenching (qI) of PSII in *C. reinhardtii* (Roach et al., 2013; Polukhina et al., 2016). The cells can use acetate contained in TAP medium to produce NADPH by catabolism and induces type II NADPH dehydrogenase (NDA2)-dependent cyclic electron flow (CEF) (Johnson and Alric, 2012; Burlacot et al., 2019), resulting in PSII limitation by over-reduction of the plastoquinone (PQ) pool in extreme photooxidative stress. This matches well with our PAM data showing very low NPQ value in overexpression mutant as well as knocked down strains. As a result, ELIP3 needs to be accumulated as the limitation was shifted back to PSII, however, qI was strongly induced by severe stress conditions despite the accumulation of ELIP3 in the absence of CO_2_ influx. The CO_2_ influx that could alleviates these limitations affected the accumulation of ELIP3 as well as D1 protein. These results suggest that the accumulation of ELIP3 affects more to qI than qE.

What is the role of ELIP3 in *C. reinhardtii*? Our results suggest that the accumulation of ELIP3 helps cell to alleviate photooxidative stress at low temperature. Then how? The function of ELIPs in higher plants is to disperse excess amounts of absorbed energy in the form of heat or fluorescence (Adamska et al., 1999; Montané and Kloppstech, 2000). ELIPs in higher plants are also known to inhibit the production of harmful ^1^O_2_ from ^3^Chl by neutralization and degradation of free chlorophyll in photosystem under stress conditions (Hutin et al., 2003; VanBuren et al., 2019). A reduction in zeaxanthin, a known NPQ agent, was observed in experiments using *Arabidopsis elip1*/*elip2* mutant, suggesting that ELIPs are associated with xanthophyll regulation too (Rossini et al., 2006). There are other proteins involved in the protection of photosystem from photooxidative stress in *C. reinhardtii.* For example, light-harvesting complex stress-related protein 3 (LHCSR3) which has similar transmembrane structure to ELIP3 regulates qE in high light stress and is known to protect photosystems (Maruyama et al., 2014; Girolomoni et al., 2019; Roach et al., 2020). Difference lies in that only ELIP3 shows low-temperature dependent expression. Western blot analysis on D1 protein in PSII offered some clue about the function of ELIP3. D1 protein degraded when PSII is suffering from photooxidative stress in low temperature. When the accumulation of ELIP3 protein was reduced by TEMPOL treatment, D1 protein was degraded even though cellular oxidative stress was reduced. The accumulation of ELIP3 appears to be affected by the superoxide anion (O_2_^–^) generated from PSI because TEMPOL scavenges only O_2_^–^ and does not remove other harmful ^1^O_2_ generated in PSII. Our results showed degradation of D1 proteins at low temperature even when cellular O_2_^–^ was scavenged with the treatment of TEMPOL. These results may suggest that some ROS signaling is required for the maintenance of D1 in low temperature, or TEMPOL may cause damage to PSII although we could not find any reference on it. It is also possible that D1 protein in PSII requires ELIP3 for protection against photooxidative stress. Norflurazon treatment increases harmful ^1^O_2_ in PSII by inhibiting carotenoids production (Larkin, 2014). It is interesting that D1 protein was not degraded with the treatment of norflurazon, while ELIP3 accumulated. A knockdown mutant of ELIP3 showed faster degradation of D1 protein than WT under photooxidative condition supporting the idea that ELIP3 may be involved in the protection of other proteins in the photosystem. ELIP3 appears to have similar function to that of ELIPs in higher plants in protecting photosystem from oxidative stress. It is noteworthy that TAP medium that we used in this study provides mixotrophic condition to *C. reinhardtii* and makes it difficult to interpret our data in relation with ELIP3 function as qE. Further studies using various media will provide more information about the function of ELIP3 in maintaining NPQ under cold stress.

Cellular ROS is not the only signal that triggers accumulation of ELIP3 in *C. reinhardtii.* Strong accumulation of ELIP3 was induced by UV-A irradiation while UV-B irradiation did not induce any accumulation suggesting that a photoreceptor-mediated regulation may be involved in its expression. Cryptochrome (CRY), a blue/UV-A photoreceptor, has been proposed as a regulator for the expression of ELIPs in higher plants (Kleine et al., 2007). A transcription factor ELONGATED HYPOCOTYL 5 (HY5) that affects CRY1-dependent high light response has been identified in *Arabidopsis ELIP2* promoter studies (Kleine et al., 2007; Hayami et al., 2015). However, not all ELIPs in higher plant are CRY-dependently induced (Kleine et al., 2007), nor regulated by HY5-type transcription factors (Hayami et al., 2015). Further functional studies using transgenic mutants of CRY and ELIP3 are necessary to elucidate the signals involved in this response in *C. reinhardtii*.

The direction of phototaxis in *C. reinhardtii* is regulated by the redox poise of the cytoplasm (Wakabayashi et al., 2011). Phosphorylation of channelrhodopsin-1 is affected by the redox state of the cytoplasm and changes with phototactic behavior in response to physiological stimuli in *C. reinhardtii* (Böhm et al., 2019). Our results showed that the knockdown mutant of ELIP3 showed negative phototaxis at room temperature suggesting that the mutant has different redox poise of the cytoplasm from that of WT or overexpression strain. *C. reinhardtii* has a homeostatic negative feedback mechanism to maintain a slightly reduced environment and to avoid oxidative damage in the cell via modulation of photosynthetic activity (Wakabayashi et al., 2011). The knockdown strain shows lower effective and maximum quantum yield than WT even in moderate light and warm temperature. These results suggest that decreased photosynthetic activity in the knock down mutant could fail to maintain a reduced environment in the cytoplasm which may result in altered direction of movement. Further studies on the signals for the phototaxis in relation with the expression of ELIP3 may reveal other roles of ELIP3 in the regulation of movement in *C. reinhardtii*.

Cold temperature is one of the most serious challenges in developing large-scale microalgal production facility outside of tropical or sub-tropical locations (Laamanen et al., 2014). Temperature often drops at night below 10°C even in temperate regions, which causes photooxidative damage to the cell. Our results show that *ELIP3* is a promising target gene to develop cold-tolerant strains because it is involved in the protection of photosystems and helps to enhance photosynthetic efficiency at low temperature. Further studies using various combination of transgenic mutants are necessary to achieve the final goal in developing useful cold-tolerant strains of *C. reinhardtii*.

## Data Availability Statement

The original contributions presented in the study are included in the article/Supplementary Material, further inquiries can be directed to the corresponding author.

## Author Contributions

JWL, SHL, and GHK conceived and designed research. JWL and SHL conducted experiments. JWL and JWH contributed research idea and analytical tools. JWL and GHK analyzed the data and wrote the manuscript. All authors read and approved the manuscript.

## Conflict of Interest

The authors declare that the research was conducted in the absence of any commercial or financial relationships that could be construed as a potential conflict of interest.

## Footnotes

1 http://www.chlamycollection.org/
