# Supplementary material for: Early Light-Inducible Protein (ELIP) Can Enhance Resistance to Cold-Induced Photooxidative Stress in Chlamydomonas reinhardtii
Source: Front Physiol. 2020 Aug 25;11:1083. doi: 10.3389/fphys.2020.01083 (PMC7478268; doi:10.3389/fphys.2020.01083)
Supplement: Supplementary file 1 [file Table_1.DOCX]

Supplementary Material

# Supplementary Figures


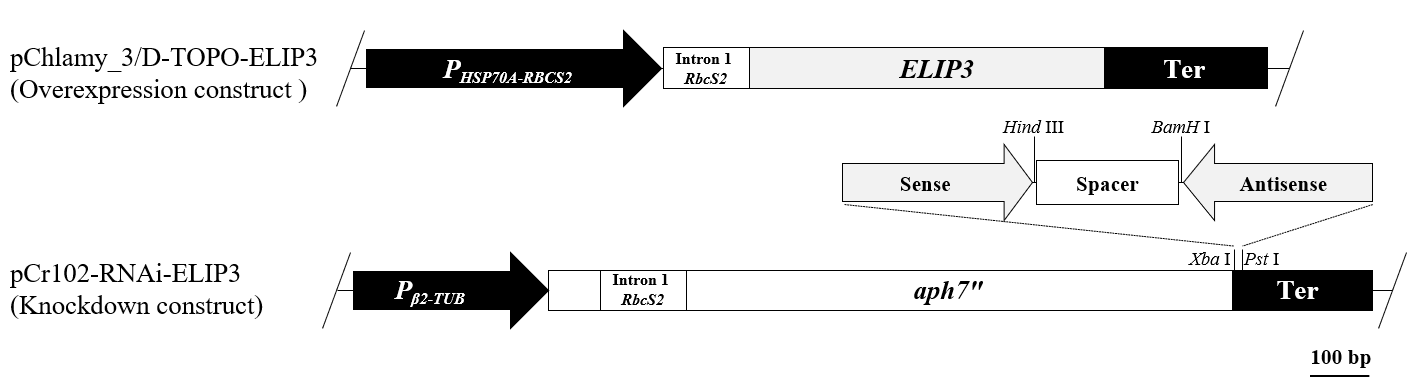


**Supplementary Figure S1.** Schematic diagram of the construct for overexpression and knockdown (RNAi) of *ELIP3*. Knockdown construct used to induce RNAi, which expresses hairpin *ELIP3* dsRNA by cloning forward and reverse partial *ELIP3* coding sequences (314 bp) on both sides of DNA spacer in the 3′ UTR of *aph7*″ marker gene (the hygromycin resistant gene).


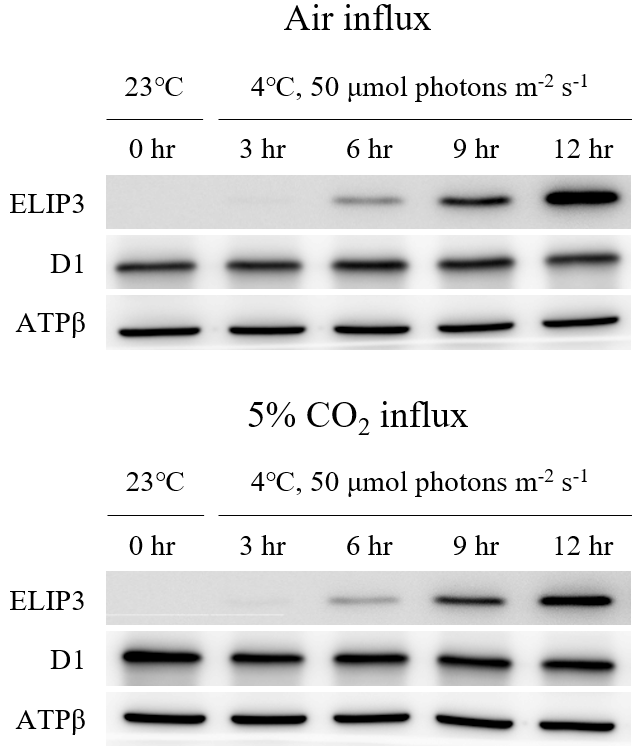


**Supplementary Figure S2.** Western blot analysis of ELIP3 and D1 in *C. reinhardtii* with or without supply of 5% CO_2_ under 4℃ and nonstress light intensity (50 μmol photons m^-2^ s^-1^). ATPβ was detected as a loading control.

# Supplementary Table

**Supplementary Table S1.** List of primers used in this study.

| Name | Sense primer (forward) | Antisense primer (reverse) |
| --- | --- | --- |
| ELIP3_RNAi | AACTGCAGATCTAGACGCCTGGCCATGCTGGGCTT | AACCTAGGAAAGCTTGCCGCAGCAGAATCGGCTGT |
| ELIP3_qPCR | GTGGCACCGTCGAG CCCAC | GCGGTGCCGCTGAAGGACAT |
| 18s rRNA_qPCR | CCTGCGGCTTAATTTGACTC | ACCGGAATCAACCTGACAAG |
| ELIP3_Northern blot | GATGGCACCGTCGAGCCCAC | GCGGTGCCGCTGAAGGACAT |
